# S112. RELATION BETWEEN EARLY-ONSET PSYCHOSIS AND FORMAL THOUGHT DISORDER IN SCHIZOPHRENIA

**DOI:** 10.1093/schbul/sby018.899

**Published:** 2018-04-01

**Authors:** Koksal Alptekin, Simge Altinok, Halis Ulas, Banu Degirmencioglu, Berna Akded

**Affiliations:** Dokuz Eylul University

## Abstract

**Background:**

Formal thought disorder is one of the fundamental features of schizophrenia. Early onset schizophrenia (EOS) is strongly related to poor prognosis and illness outcomes. The aim of this study is to investigate the relation of EOS and formal thought disorder in schizophrenia.

**Methods:**

This research was a retrospective study. Data regarding the patients with schizophrenia were obtained from two separate studies conducted at Dokuz Eylul University.

Thought disorder scores were compared between 32 patients with early onset schizophrenia (EOS; age ≤ 18) and 120 patients with adult onset schizophrenia (AOS; age > 18). Also, we looked at the effect of Duration of Untreated Psychosis (DUP). We further categorized these two sets as short DUP (short DUP; ≤ 6 month) and long DUP (long DUP; > 6 month) groups.

**Results:**

Schizophrenia patients with early onset showed significantly higher scores compared to adult onset schizophrenia patients with regards to poverty of speech (U= 1525.50; p = .037) and peculiar sentences (U= 1613.50; p = .043).

**Discussion:**

Early onset schizophrenia patients had significant formal thought disorder abnormalities. Formal thought disorder may have some developmental characteristics which indicate an important dimension related to prognosis and outcome of schizophrenia. Also, DUP may have potential effect over formal thought disorder.

